# Multiple enchondromatosis (Ollier disease)

**DOI:** 10.5144/0256-4947.2009.65

**Published:** 2009

**Authors:** Suhil A. Choh, Naseer A. Choh

**Affiliations:** a From the Department of Pediatrics and Neonatology, Sher-i-Kashmir Institute of Medical Sciences, Srinagar Jammu and Kashmir, India; b From the Department of Radiology SMHS Hospital, Srinagar Jammu and Kashmir, India

A19-year-old male patient, a product of a non-consanguineous marriage, presented to our outpatient clinic with a 5-month history of painless swelling of the fingers of both hands and a right-sided limp during the previous 2 months. The general physical examination was unremarkable except for swelling of the fingers. Routine lab investigations were normal. Radiographs showed radiolucent lesions of fingers, pelvis and lower end of the right femur, which were diagnosed as enchondromas.

Enchondromas are benign cartilaginous tumors that develop in the metaphysis and may become in-corporated into the diaphysis in close proximity to the growth plate cartilage. Typically the tumors involve the short bones of the hands and feet.^1^ Multiple enchondromatosis with a predominantly unilateral distribution is referred to as Ollier disease.^2^ Ollier disease is characterized by an asymmetric distribution of the lesions and an extreme clinical variability.^2^ Clinical manifestations include painless asymptomatic palpable bony masses of the phalanges or toes, pathological fractures, limb-length discrepancy with limping and the potential risk for malignant change to chondrosarcoma.^2^,^3^

Ollier disease is not genetically determined in a simple Mendelian manner. Apart from the short tubular bones of the hand and feet, enchondromas frequently affect the long tubular bones, the flat bones and pelvis. The lesions are multiple and asymmetrically distributed, predominantly affecting one side of the body. Malignant transformation, reported in 15% to 25%, is clinically suspected by pain and a rapid increase in the size of the lesions.^4^ A biopsy is needed to demonstrate sarcomatous degeneration. The diagnosis of Ollier disease is based on clinical features and radiographs that show multiple radiolucent lesions in the metaphysic.^2^,^5^–^7^ The lesions run parallel to the long axis of bone.

**Figure 1:**
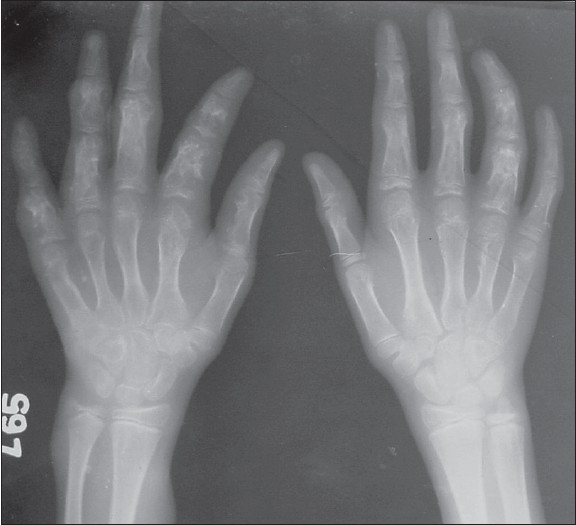
Radiograph showing radiolucent expansile lesions involving the metacarpals and phalanges bilaterally.

**Figure 2 and 3:**
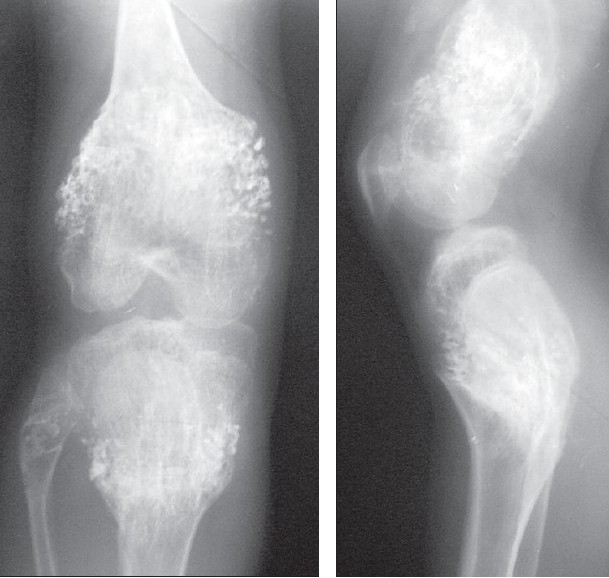
Anteroposterior and lateral radiographs showing multiple columnar cartilaginous masses of the right lower end of the femur and upper end of the tibia and fibula.

**Figure 4:**
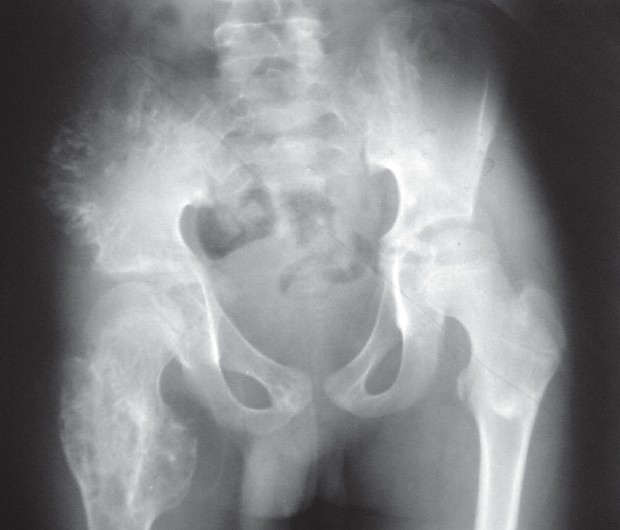
Anteroposterior radiograph of the pelvis showing columnar, radiolucent lesions in the right ilium extending to the crest and suggestive of cartilaginous rests. Similar lesions are seen in the greater trochanter of the right femur.

Tubular bones in the hands are expanded by the lesion and become globular. Enchondromas are originally localized to the growth plate cartilage, and progressively migrate towards the diaphysis. It should be emphasized that enchondromatosis has a mostly unilateral monomelic distribution. If the entire body is involved, one half is more affected.^2^,^3^,^7^ Solitary lesions are treated with curettage and bone grafting. The treatment of multiple enchondromatosis should be tailored to the lesions involving the most severely affected limb.
